# Salivary Biomarkers: Noninvasive Ways for Diagnosis of Parkinson's Disease

**DOI:** 10.1155/2023/3555418

**Published:** 2023-07-03

**Authors:** Sanaz Salaramoli, Hamid Reza Joshaghani, Seyed Isaac Hashemy

**Affiliations:** ^1^Student Research Committee, Faculty of Medicine, Mashhad University of Medical Sciences, Mashhad, Iran; ^2^Department of Clinical Biochemistry, Faculty of Medicine, Mashhad University of Medical Sciences, Mashhad, Iran; ^3^Laboratory Sciences Research Center, Golestan University of Medical Sciences, Gorgan, Iran

## Abstract

Finding reliable biomarkers has a crucial role in Parkinson's disease (PD) assessments. Saliva is a bodily fluid, which might be used as a source of biomarkers for PD. Our article has reviewed several publications on salivary proteins in PD patients and their potential as biomarkers. We find out that *α*-Syn's proportion in oligomeric form is higher in PD patients' saliva, which is potent to use as a biomarker for PD. The salivary concentration of DJ-1 and alpha-amylase is lower in PD patients. Also, substance P level is more moderate in PD patients. Although salivary flow rate is decreased in PD patients, high levels of heme oxygenase and acetylcholinesterase might be used as noninvasive biomarkers. Salivary miRNAs (miR-153, miR-223, miR-874, and miR-145-3p) are novel diagnostic biomarkers that should be given more attention.

## 1. Introduction

Parkinson's disease (PD), the second most common progressing neurodegenerative disorder, concerns about 2% of middle-aged people [1]. PD symptoms contain impaired motor functions with slow movements and tremors. Also, patients encounter autonomic malfunctions with hypotension, defecation, urinary, and sleep disorders [2]. PD is identified by losing dopaminergic neurons in the substantia nigra pars compacta (SNpc), deposition and aggregation of alpha-synuclein (*α*-Syn) into Lewy bodies, and neurites or oxidative stress [3, 4]. Besides, glial neuroinflammatory reactions are contributed to degenerative procedures in PD [5]. Moreover, pieces of genetic research point to the role of mutations in coding SNCA genes (*α*‐Syn) in this neurodegeneration [6, 7]. However, no reliable diagnostic biomarker exists for PD yet, its diagnosis is usually clinical, and an autopsy is needed for verification [8]. Thus, diagnosing and prognosing biomarkers for PD remained a primary unmet demand. Biomarkers help identify targets for treatment, and they are vital to understanding the PD pathophysiology [9, 10]. There are four classifications of biomarkers: clinical, imaging, biochemical, and genetic [11]. Body liquids, such as blood, urine, and cerebrospinal fluid, have traditionally been used for biomarker analysis due to their accessibility and the presence of a wide range of biomolecules. However, these samples can be challenging to collect, require specialized equipment and expertise for processing, and can be invasive, which may limit their utility in certain clinical contexts [12]. In recent years, the potential use of saliva as a biomarker source has gained attention due to its noninvasive collection method, lower risk of infection, and potential for point-of-care testing. Saliva contains a diverse array of biomolecules, including proteins, nucleic acids, and metabolites that can provide valuable information about oral and systemic health, making it a promising source for biomarker analysis [13]. Furthermore, saliva is highly stable and readily stored, transported, and analysed, thereby enabling convenient and cost-effective biomarker characterization. Hence, salivary biomarkers hold tremendous potential for transforming clinical diagnostics and personalized medicine by providing simple, noninvasive, and accurate detection and monitoring of disease states [14]. Saliva has emerged as a promising body fluid for obtaining samples for PD diagnosis due to its noninvasive collection method, lower risk of infection, and potential for point-of-care testing [15].

Here, we review recent developments in biochemical biomarkers, considering their advantages and limits for PD diagnosis. The biochemical biomarkers included genes, RNAs, microRNAs, proteins, peptides, and neurotransmitters which all are presented in Table 1 as a summary [44, 45].

## 2. Alpha-Synuclein

Alpha-synuclein (*α*-Syn), one of the major components of Lewy bodies [46], predominantly express in the neocortex, hippocampus, SNpc, thalamus, and cerebellum regions of brain [47]. *α*-Syn is encoding by the SNCA gene [48], consisting of 140 amino acids with 14 kDa molecular weight [49]. As remarked, the dominant form of *α*-Syn is the full-length protein, and its C-terminal truncations cause aggregation and suggest that C-terminal alterations may be involved in *α*-Syn pathology [50]. Despite important research, the structure of *α*-Syn is not determined precisely. It has been defined as intrinsically disordered, helical, or a combination of both [51], in which, Helix-rich structure is much more reliable according to the presence of phospholipid membranes and protein's function [52].

*α*-Syn is abundantly expressed in the brain, particularly in presynaptic nerve terminals. While its precise function is not fully understood, *α*-Syn is thought to play a role in synaptic plasticity, vesicle trafficking, and regulation of neurotransmitter release. In healthy cells, *α*-Syn exists as a soluble monomer, but it can undergo a conformational change and aggregate into insoluble fibrils, which are a hallmark of several neurodegenerative diseases, including PD and Lewy body dementia. The mechanisms underlying *α*-Syn aggregation and toxicity are complex and not fully understood, but some evidence suggests that it may be linked to impaired proteasomal and lysosomal function, oxidative stress, and inflammation. Therefore, the biological role of alpha-synuclein may have implications for the development of novel therapeutic strategies for neurodegenerative diseases [53].

As mentioned above, the pathological hallmark of PD is *α*-Syn aggregation within the central nervous system (CNS) neurons. Also, evidence has proven that misfolded *α*-Syn accumulates in the peripheral tissues, for example, in the early stages of the gut disease, providing the display of the peripheral immune system to CNS antigens [54, 55]. There is plenty of monomeric *α*-Syn in presynaptic terminals of neurons in the CNS [56]. The protein species contain a wide range, from soluble oligomers to insoluble fibrillar forms [57]. Many researchers have attempted to study *α*-Syn in serum as a biomarker that its alteration might be a distracting factor in *α*-Syn antibodies measurements because they may be undetectable if already bound together [58]. On the other hand, *α*-Syn antibodies may have a protective role, promoting the clearance of toxic protein through the opsonization of *α*-Syn for uptake by phagocytes [59]. Aggregated *α*-Syn (oligomeric and phosphorylated) forms exist in body fluids including cerebrospinal liquid (CSF), serum/plasma, urine, and saliva, and in gastrointestinal tract, vagus nerve, sympathetic ganglia, cutaneous autonomic nerves, and submandibular gland [8], but data for this neurological protein, as a validated biomarker to predict or diagnose PD in human body fluids, are limited [16]. Besides, publications on CSF and plasma/serum *α*-Syn levels have controversial outcomes due to preanalytical/analytical factors such as total/oligomeric forms of *α*-Syn [60].

On the other hand, although there are several procedures, such as ELISA, western blot, mass spectrometry, or Luminex assay, to discover *α*-Syn in body fluids [61]; neither plasma/serum nor saliva *α*-Syn has achieved a proper sensitivity or specificity to be verified as a PD diagnostic marker [17, 18]. Thus, the most important subsequent investigation in the PD biomarkers researching is improving sensitivity and specificity assays of available markers [16].

As before mentioned, *α*-Syn exist in body fluids such as saliva. Collecting saliva is exclusively simple, noninvasive, and nonaffected by blood contamination [62]. In addition, saliva is a more stable sample than CSF, which is prone to degradation over time and requires immediate storage and processing [19]. Furthermore, saliva has been shown to contain *α*-Syn in a similar concentration to CSF, and studies have demonstrated a significant correlation between *α*-Syn levels in saliva and in CSF [60]. Devic et al. had investigated that available *α*-Syn in saliva is potential to be a PD biomarker [20]. Their study prompted that the huge amount of saliva is produced in the human submandibular glands [21], which are affected in the early stages of PD through synucleinopathy [63]. After that, Kang et al. had studied levels of total salivary *α*-Syn by using Luminex assay and oligomeric *α*-Syn by the combination of two chromatography and western blot methods. Their study described that total *α*-Syn proportions in saliva could be manipulated by various *α*-Syn single nucleotide polymorphisms (SNPs) and are not sufficient to be used as a unique biomarker. Still, salivary oligomeric *α*-Syn can be a probable diagnostic marker for PD [22]. Another study has demonstrated that the mixed detection methods of salivary total and oligomeric *α*-Syn might help the early diagnosis of PD. They claimed that concentration of total *α*-Syn is lower in saliva, whereas oligomeric *α*-Syn concentration is higher in PD patients compared to wholesome participants (using ELISA technique). The oligomeric/total *α*-Syn ratio is also higher in patients compared to controls. Total *α*-Syn proportion in saliva is associated negatively with oligomeric *α*-Syn and positively with patients' clinical features [23].

In PD, a reduction of total *α*-Syn reflects reducing *α*-Syn monomers concentration and forming both intracellular insoluble and soluble compositions [24]. A few years later, In order to maintain the previous results, they claimed that detecting *α*-Syn in saliva is a favourable biomarker for PD [64]. It was suggested that the differences in total and oligomeric *α*-Syn concentrations are attributed to the salivary monomer *α*-Syn oligomerization, leading to a decrease in total *α*-Syn concentration. Moreover, a raised oligomeric *α*-Syn proportion was previously found in both plasma and CSF in PD patients [26, 65, 66]. Similarly, much research has shown the same results; they claimed that salivary *α*-Syn level in PD subjects is considerably less than in healthy participants. No correlation has been found between *α*-Syn concentration and motor/nonmotor symptoms in PD subjects [25, 67]. Another study evaluated the concentrations of various salivary *α*-Syn forms, including total *α*-Syn, oligomeric *α*-Syn, and *α*-Syn PS129 to estimate the specificity and sensitivity *α*-Syn in detecting PD [68]. Both oligomeric *α*-Syn and oligomeric *α*-Syn/total *α*-Syn ratio were more elevated in PD than healthy subjects. Besides, no differences were found in proportions of total *α*-Syn, *α*-SynPS129, or ratio of *α*-SynPS129/total *α*-Syn between PD and control subjects. The oligomeric *α*-Syn differentiated PD levels from healthy subjects with around 90% sensitivity and specificity, and oligomeric *α*-Syn/total *α*-Syn distinguished between PD and healthy subjects with about 80% and 70% of sensitivity and specificity, respectively [68]. Besides, research performed by Shaeen et al, revealed that both total and oligomeric *α*-Syn in saliva is considering being potential biomarkers to diagnosing PD; what stands out from their given data is that oligomeric *α*-Syn level and oligo/total ratio increase among PD patients, while total proportion decreases significantly. However, a noticeable increase in oligomer concentration was found in patients with bradykinesia and rigidity symptoms [69]. In this regard, a recently published meta-analysis article on salivary *α*-Syn has demonstrated a significant difference in the proportion of *α*-Synuclein forms (total, oligomer, and oligomer/total) between PD and control subjects' saliva [70].

In contrast, Goldman et al., in a cohort study of moderately advanced PD, demonstrated that the concentration of *α*-Syn in saliva is the same between PD and healthy participants by analysis of clinical data and specimens, such as body fluids. Also, they did not find notable association between salivary *α*-Syn, CSF, and plasma or even between *α*-Syn in saliva and patients' motor symptoms [71]. In another research on cheek cell-derived *α*-Syn, no differentiation was detected in PD and healthy ones. Furthermore, there was no association between the concentration of *α*-Syn, age, and sex and no associations between *α*-Syn concentration and motor functions in PD subjects [72].

Since, discriminating multiple system atrophy-Parkinsonism MSA is challenging, Cao et al. study suggested salivary *α*-Syn could help efficiently distinguish MSA from PD. In saliva, the total *α*-Syn concentrations were lower in MSA than PD. No significant difference was shown in oligomeric *α*-Syn and *α*-Syn PS129. Total *α*-Syn 4.46 pg/ng distinguished MSA from PD with area under the curve (AUC) 0.804 [73]. Luan et al, even demonstrated that salivary *α*-Syn seeding activity may serve as a novel biomarker for the clinical diagnosis of PD and MSA. They claimed that salivary *α*-Syn RT-QuIC assay distinguished patients with PD. In their study, no significant differences were observed in the diameter of salivary *α*-Syn fibrils examined by electron microscopy and in thioflavin T fluorescence intensity of salivary *α*-Syn fibrils detected by RT-QuIC assay between patients with PD and MSA. Notably, the lag phase of RT-QuIC assay from patients with PD was significantly shorter than that of patients with MSA, which might be clinically applicable to the discrimination between PD and MSA [74].

In addition to saliva, urinary *α*-Syn has been noted to researchers nowadays but there are limited studies using urine to evaluate *α*-Syn as a biomarker. Nam et al. demonstrated a notable decrease in levels of urinary Fila-*α*-Syn and total *α*-Syn in PD patients in comparison with non-PD subjects [75]; moreover, urinary Fila *α*-Syn/total *α*-Syn ratio was moderately elevated in PD patients, which may be used as PD biomarker [76].

## 3. DJ-1

DJ-1 is known as Parkinson disease protein 7 (PARK7 in the brackets) or an oxidative stress sensor [77]. Firstly, the DJ-1 (PARK7) gene has been recognized as a new oncogene [27, 78]. Then, finding a deletion and a missense mutation of the DJ-1 gene in PD resulted in introducing the DJ-1 gene as a possible candidate gene for familial PD [28]. The protein of DJ-1 contains 189 amino acids with seven beta-strands and nine *α*-helixes, presenting as dimers [79, 80] one *α*-helix at the C-terminal region and blocking the DJ-1 catalytic site [29]. However, the activation of DJ-1 protein is a complex process that is not fully understood. DJ-1 is involved in the regulation of various transcription factors, including Nrf2, PI3K/PKB, and p53, to protect cells against oxidative stress. Nrf2 is a stress-activated transcription factor that plays a role in protecting cells against oxidative stress and metabolic pathways by initiating NADPH and ATP production. DJ-1 promotes Nrf2 nuclear translocation and binding to antioxidant response elements by inducing the dissociation of Nrf2 from its inhibitor Keap1. DJ-1 is also a coactivator of the transcription factor NF-kB. Under nitrative stress, DJ-1 inhibits phosphatase activity by regulating PI3K/PKB signalling through PTEN transnitrosylation. DJ-1 has a complex effect on the p53 pathway, as it can both bind to p53 to restore its transcriptional activity and stimulate deacylation to suppress p53 transcriptional activity. These effects of DJ-1 on the activation of various transcription factors and redox balance help to protect neurons against the aggregation of a-syn and oligomer-induced neurodegeneration [81, 82]. Furthermore, studies have shown that DJ-1 mutants, including M26I, L166P, and D149A, exhibit diminished neuroprotective and transcriptional coactivator properties. This indicates that DJ-1 plays a role in preventing neuronal apoptosis by regulating oxidative stress and gene expression in neurons. This information could contribute to a better understanding of the biological function of DJ-1 in the pathogenesis of PD [83].

DJ-1, as a multifunction protein, joins in transcriptional regulations [83], antioxidant reactions [84] and chaperones [30], proteases [29], and mitochondrial regulations, which are expressed in all cells and tissues, particularly in both brain neurons and glial cells [31]. Also, DJ-1 in body fluids has previously been investigated as a possible biomarker of PD in disorders with accumulated *α*-Syn [32, 85].

In addition to *α*-Syn, DJ-1 can be used as a diagnostic biomarker of PD because as *α*-Syn concentrations tend to diminish, DJ-1 levels grow in PD [20]. Masters et al.'s study claimed that the difference between PD patients' saliva and healthy subjects supports the idea that saliva is an excellent specimen for PD diagnosis. One of the distinctive differences is that salivary glands can be a significant source of additional salivary DJ-1 protein [86]. Moreover, a cohort study has investigated that salivary DJ-1 is potent to be a prognostic marker to evaluate nigrostriatal dopaminergic functions in PD subjects by finding the association of salivary DJ-1 levels and putamen nucleus, which uptakes the labelled dopamine transporters [33]. Since the alterations of DJ-1 expression are specifically vital in saliva, an electrochemical-based neurobiosensor system has been developed to detect the salivary DJ-1. Its selective determination range is 4.7–4700 fg mL^−1^ following the charge transfer resistance (Rct) associated with limited detection of 0.5 fg mL^−1^ [34]. Among all the findings, Stewart et al. study has shown an age-dependent accumulation of DJ-1 levels present in male PD patients, but no association was found between DJ-1 concentration and age in females [72].

Beside salivary DJ-1, the proportion of DJ-1 in urine is more elevated (1.7-fold) in an age-dependent manner in male PD compared to non-PD. Thus, Urinary DJ-1 can be used as a clue leading to a novel marker to detect PD, at least in males [87].

## 4. Substance P

Hyper-salivation is an early symptom of PD, while saliva production is typically diminished. A higher frequency of spitting in PD patients indicates that inadequate and irregular swallowing rather than raised saliva production is responsible for hyper-salivation [88]. Thus, substance P (SP), a neuropeptide (belongs to tachykinin family, encoded by TAC1), is known to enhance the swallowing, which is released from the hypothalamus, substantia nigra, and spinal cord [35, 36, 89], might have a beneficial role in PD prognosis. Ebihara et al.'s study has shown that SP concentration in sputum significantly decreases in advanced PD patients with impairment of both the motor and sensory components of cough [90]. Another study has shown that salivary SP concentrations are considerably less in PD with dysphagia (altered swallowing) than without dysphagia [91]. Similarly, Troche et al. demonstrated that in progressive PD stages, there is a reduction in SP level in the sputum, resulting in disruption of defensive reactions, including cough, swallowing, and sequentially silent aspiration [92]. On the other hand, decreased salivary SP levels may prognosis pharyngeal swallowing dysfunctions in PD. So, an impairment in SP-mediated neurotransmission might have notable effects on developing dysphagia in PD [93]. Despite these several studies' results, more extensive research is demanded to verify SP as a potential marker for PD-related dysphagia's prognosis.

## 5. Heme Oxygenase-1

Heme oxygenase-1 (HO-1), a 32 kDa stress-responsive protein, is a postulated oxidative stress biomarker in PD, indicating the adaptive response of human body adaptive response to raised ROS proportions in PD [94]. HO-1 is involved in the breakdown of heme into biliverdin, iron, and carbon monoxide and plays a crucial role in maintaining cellular homeostasis and protecting cells from oxidative damage [95]. In recent years, a growing body of evidence has suggested that HO-1 may serve as an early diagnostic marker for a range of pathological conditions. For instance, studies have demonstrated that HO-1 expression is upregulated in response to cellular stress and injury, making it a potential indicator of disease onset or progression [96]. In addition, HO-1 has been implicated in the regulation of inflammation, angiogenesis, and immune function, all of which are key processes involved in a variety of diseases. As such, HO-1 may hold promise as a valuable biomarker for early diagnosis and monitoring of diseases in various clinical settings [37]. Galindez et al. reported that combination of HO-1 level in with covariates can be used as a distinguishing PD marker [38]. Moreover, another investigation compared the concentration of salivary HO-1 in PD subjects with various disease severities. They have reported that salivary HO-1 concentration was more elevated in PD compared to control subjects. Also, its proportion was associated with the H&Y scores in which the level of HO-1 was higher in the early grades than in grade 2 or grade 3 which its proportion was not dependent on age, gender, comorbid disorders, and medicine exposure [39].

## 6. Acetylcholinesterase

Although PD is accompanied by nigrostriatal dopaminergic loss, recent investigations are emphasized features caused by loss of cholinergic neurons in PD [40]. Because cholinergic neurons synthesize Acetylcholinesterase (AChE) specifically, AChE has been suggested as a possible biomarker of cholinergic action [41]. The usage of salivary AChE as a PD marker results from observing xerostomia and decreasing saliva as concomitant symptoms of the disease [97]. In research done by Fedorova, PD subjects had significant growth of AChE activity/total protein (TP) ratio in saliva (AChE always be combines with TP), followed by reducing the salivary flow rate. Nevertheless, the concentration of AChE and the ratio of AChE/TP is not associated with the Unified PD Rating Scale (UPDRS) scores. In addition, no association was found between salivary AChE activities and various PD stages [97]. In line with this study, another research conducted by Pawlik et al. has shown the same results. Notably, they have demonstrated AChE/TP ratio is considerably raised in PD, suggesting that elevated activity of AChE cannot be introduced just by an up proportion of saliva. The UPDRS score presented a considerable association with TP and saliva flow rate [23]. However, due to the limited papers we could find, latter research is required to clarify if salivary AChE reacts to the degree of neuronal damage in PD patients.

## 7. Alpha-Amylase

Salivary alpha-amylase is a reliable marker for stress-response alterations in human body, reflecting the sympathetic nervous system (SNS) activities [98]. It is a slight considered marker but an excellent candidate for next studies. However, psychological stress is reported to induce salivary alpha-amylase secretion via the SNS. Since PD is caused by an impairment in the autonomic nervous system [42, 43], stress-induced salivary alpha-amylase is reduced in PD in comparison with other neurodegenerative disorders [99]. On the other hand, as a simple and noninvasive procedure, salivary alpha-amylase can improve the prognosis of SNS dysfunction in PD subjects and provides valuable diagnostic data to differentiate PD from other neurodegenerations [100].

## 8. Cortisol

Stress is associated with increasing cortisol level and inflammatory response [101]. Obviously, stress has a key role in promoting PD [102]. So, stress responses are related to motor/nonmotor symptoms which are the possible factors to diagnosis [103]. However, there are so many contradictory results on salivary cortisol concentration in PD patients. Two investigations have shown that salivary cortisol proportion is less in PD patients, linked to their antisocial behaviour. However, there is no considerable correlation between salivary cortisol levels and age, sex, BMI, using levodopa, or PD severity [104, 105]. Elevated salivary bedtime cortisol has been reported among PD patients [106]. In contrast, research by Skogar et al. claimed that the neurological alterations in PD do not interrupt the hypothalamic-pituitary-adrenal axis. Thus, cortisol levels in PD patients' saliva will increase every morning, but it is not influenced by motor dysfunctions, disorder period, or simultaneousness of pain [107]. However, a pilot study suggests that association of anxiety and depression with increased salivary cortisol levels may be involved in neuroendocrine alterations in PD pathophysiology [108].

## 9. MicroRNAs

Since microRNAs (miRNAs) discovery, miRNAs have been significant biological molecules involved in cellular functions [109]. Many studies have reported that miRNAs implicate several neurodegenerative disorders, including PD miRNAs also have a key role in regulating the posttranscriptional expression of several genes, including DJ-1 or *α*‐syn. [110]. Such known miRNAs involved in *α*‐Syn regulation are named: miR‐153 and miR‐223 which they had experienced a downregulation in the brain, serum, and saliva of Parkinson's disease GFAP.HMOX1 transgenic mice model. Thus, miR‐153 and 223 in saliva can preserve as valuable and noninvasive markers to diagnose idiopathic PD with 79% and 77% accuracy, respectively [111]. Moreover, salivary miR-874 (with 64.3% sensitivity and 78.6% specificity) and miR-145-3p (with 60.0% sensitivity and 75.0% specificity) expression, regulating the expression of DJ-1 gene, are more elevated in PD than healthy subjects without any apparent correlation to age or gender. Therefore, the salivary expression of miR-874 and miR-145-3p in saliva can also be introduced as an additional biomarker for PD [112]. Jiang et al., found 34 differential miRNAs in PD patients' saliva which may be implicated in the pathogenesis of the disease and in molecular functions such as ubiquitin protein ligase activity. They identified three differentially expressed miRNAs (miR-29a-3p, miR-29c-3p, and miR-6756-5p). The combination of salivary miR-29a-3p and miR-29c-3p has potential to be a noninvasive diagnostic biomarker for idiopathic PD, and miR-29a-3p may be useful for differentiating PD from ET and MSA [113].

Since there is no other study on all salivary miRNAs, more extensive research is demanded on other miRNAs, which their role have been proved in serum or plasma of PD patients, such as miR-216a [114], miR-375 [115], miR-373, miR-30c-5p [116], and miR-7 [117].

## 10. Conclusion

Saliva is considered to be the optimal sample for analysing biomarkers due to its ease of collection, stability, and correlation with systemic biomarkers. Besides, it is clear that a blood-contaminated specimen might result in false increase in levels of DJ-1 and *α*-Syn [62]. According to the available data on the existing proteins in saliva involved in PD, oligomeric *α*-Syn is the best candidate biomarker for this neurodegenerative disorder. The oligomeric *α*-Syn seems crucial in the neurodegeneration process, forming nonsoluble fibrils in PD. The proportion of oligomeric *α*-Syn has been described as being elevated in the patients' saliva with idiopathic PD in comparison to healthy subjects shown as a possible PD marker. Other proteins involved in PD, including DJ-1, succeed in distinguishing PD from healthy people. Also, alpha-amylase, heme oxygenase, acetylcholinesterase, and cortisol levels can be used as a salivary marker to distinguish PD from other neural disorders or even as an early diagnostic biomarker. Besides, a neuropeptide called substance P found in saliva and sputum has been reported as a potential biomarker that differentiates PD from healthy subjects. Alongside, findings from several studies describe different miRNAs, including miR-153, miR-223, miR-874, and miR-145-3p, which are potent to be used as a PD diagnosis marker, but further studies are needed for confirmation.

Altogether, further investigations on salivary biomarkers to diagnose or prognose PD should be attending to validating diagnostic techniques based on saliva.

## Figures and Tables

**Table 1:** Biomarkers' alteration in PD.

Biomarkers	Technique	Result	Ref.
*α*‐syn	—	Present	[16]
*α*-Syn total	Luminex assay	—	[17]
Oligomeric *α*-Syn	Gel filtration chromatography/western blot	↑	
Total Α-Syn	ELISA	↓	[18]
Oligomeric *α*-Syn	ELISA	↑	
Oligo/total *α*-Syn	ELISA	↑	
Total *α*-Syn	ELISA	↓	[19]
Oligomeric *α*-Syn	ELISA	↑	[20]
Oligomeric *α*-Syn	ELISA	↑	[21]
Total Α-Syn	ELISA	↓	[22]
Oligomeric *α*-Syn	Electrochemiluminescence	↑	[23]
Oligo/total *α*-Syn	Electrochemiluminescence	↑	
Total *α*-Syn	Electrochemiluminescence	_	
*α*-SynPS129	Electrochemiluminescence	_	
Oligo *α*-Syn	ELISA	↑	[24]
Oligo/total *α*-Syn	ELISA	↑	
Fila-Syn/total Α-Syn	ELISA	↑	[25]
Total Α-Syn	Luminex assay	_	[26]
Total *α*-Syn	Mass spectrometry	↓	[27]

DJ-1			
DJ-1	Luminex assay	↑	[26]
DJ-1	Mass spectrometry	↑	[27]
DJ-1	Immunoblotting	↑	[28]
DJ-1	Western blotting	↑	[29]

Substance P			
Substance P	Radioimmunoassay	↓	[30]
Substance P	ELISA	↓	[31]
Substance P	ELISA	↓	[32]

Heme oxygenase-1			
Heme oxygenase-1	ELISA	↑	[33]
Heme oxygenase-1	ELISA/western blotting	↑	[34]

Acetylcholinesterase			
Acetylcholinesterase	Colorimetric method	↑	[35]
Acetylcholinesterase	Colorimetric method	↑	[36]

Cortisol			
Cortisol	ELISA	↑	[37]
Cortisol	ELISA	↓	[38]
Cortisol	Luminescence immunoassay	↑(morning)	[39]
Cortisol	Radioimmunoassay	↑(morning)	[40]
Cortisol	—	↑	[41]

MicroRNA			
miR-153	q-PCR	Downregulated	[42]
miR‐223	q-PCR	Downregulated	
miR-784	q-PCR	Upregulated	[43]
miR-145-3p	q-PCR	Upregulated	

## Data Availability

This review article does not contain original data. The data sources used in this review are publicly available and referenced accordingly in the article. Any additional information or data used in this review can be obtained by contacting the corresponding author.
